# Intrarater and interrater agreement and reliability of vestibular evoked myogenic potential triggered by galvanic vestibular stimulation (galvanic-VEMP) for HTLV-1 associated myelopathy testing

**DOI:** 10.1371/journal.pone.0204449

**Published:** 2018-09-27

**Authors:** Júlia Fonseca de Morais Caporali, Ludimila Labanca, Kyonis Rodrigues Florentino, Bárbara Oliveira Souza, Denise Utsch Gonçalves

**Affiliations:** Programa de Pós-Graduação em Infectologia e Medicina Tropical, Faculdade de Medicina da Universidade Federal de Minas Gerais, Belo Horizonte, Minas Gerais, Brazil; University of Minnesota College of Veterinary Medicine, UNITED STATES

## Abstract

**Background:**

The vestibular evoked myogenic potential triggered by galvanic vestibular stimulation (galvanic-VEMP) has been used to assess the function of the vestibulospinal motor tract and is a candidate biomarker to predict and monitor the human T-cell lymphotropic virus type 1 (HTLV-1) associated myelopathy (HAM). This study determined the agreement and reliability of this exam.

**Methods:**

Galvanic-VEMP was performed in 96 participants, of which 24 patients presented HAM, 27 HTLV-1-asymptomatic carriers, and 45 HTLV-1-negative asymptomatic controls. Galvanic vestibular stimulation was achieved by passing a binaural and bipolar current at a 2 milliamperes (mA) intensity for 400 milliseconds (ms) between the mastoid processes. Galvanic-VEMP electromyographic wave responses of short latency (SL) and medium latency (ML) were recorded from the gastrocnemius muscle. Intrarater (test-retest) and interrater (two independent examiners) agreement and reliability were assessed by standard error of measurement (SEM), coefficient of repeatability (CR), intraclass correlation coefficient (ICC), and Kappa coefficient.

**Results:**

In the total sample (n = 96), SL and ML medians were 56 ms (IQR 52–66) and 120 ms (IQR 107–130), respectively. The intrarater repeatability measures for SL and ML were, respectively: SEM of 6 and 8 ms; CR of 16 and 22 ms; ICC of 0.80 (p<0.001) and 0.91 (p<0.001); and a Kappa coefficient of 0.53 (p<0.001) and 0.82 (p<0.001). The interrater reproducibility measures for SL and ML were, respectively: SEM of 3 and 10 ms; CR of 8 and 27 ms; ICC of 0.95 (p<0.001) and 0.86 (p<0.001); and a Kappa coefficient of 0.77 (p<0.001) and 0.88 (p<0.001).

**Conclusion:**

Galvanic-VEMP is a reliable and reproducible method to define the integrity of the vestibulospinal tract. Longitudinal studies will clarify its validity in the clinical context, aimed at achieving an early diagnosis and the monitoring of HAM.

## Introduction

The vestibular evoked myogenic potential triggered by galvanic vestibular stimulation (galvanic-VEMP) is an exam that assesses the function of the vestibulospinal motor tract [1] and has been used as an auxiliary tool in spinal cord diseases caused by tumor, trauma, and infection [2–5]. In human T-cell lymphotropic virus type 1-associated myelopathy (HAM), galvanic-VEMP disclosed an electrophysiological altered response that ranged from a delayed latency among the asymptomatic carriers to a complete absence of response in those with established myelopathy [4].

HAM is an insidious and irremissible meningomyelitis that affects 1–4% [6–10] of the 5–20 million people infected with HTLV-1 worldwide [11, 12]. This neurologic disease is more frequent in women than in men (2:1 to 3:1), and its symptoms onset is most often found in the fifth decade of life [13–17]. The first symptoms of HAM are weakness of the lower limbs, lumbar pain, dizziness, and urinary and sexual impairments [17, 18–21]. Sensory changes may also be an early complaint [22]. The progression is characterized by spastic paraparesis and lower limb hyperreflexia, Babinski sign, impaired vibratory sensitivity, positive Romberg test, and abnormal gait. After 10 years of symptoms, 20–50% of individuals with HAM become wheelchair-dependent [19, 23–25]

HAM occurs due to an unbalanced inflammatory response to HTLV-1 infection [26–29]. The disease has a biphasic pathology pattern [30] in which the inflammatory phase is followed by an atrophic stage. The therapeutic strategies have been developed based mainly on inflammation control in the first phase, since irreversible neuron damage characterizes the later periods of the disease. Thus, the earlier the diagnosis, the better the chance of obtaining a good response to treatment [31]. In this scenario, along with the immunologic molecules, the neurophysiology exams, such as galvanic-VEMP, are candidate biomarkers to predict HAM in its subclinical stage and monitor the disease activity during treatment [4].

Vestibular evoked myogenic potential (VEMP) is an electrophysiological test in which a stimulus is offered to the vestibular system, triggering several interconnected motor responses comprising ocular and postural muscles. In VEMP triggered by galvanic vestibular stimulation (GVS), an electric stimulus is applied to the labyrinth organs through surface electrodes positioned behind the ears. The stimulus generates a dipole between the labyrinths, which is interpreted by the central nervous system (CNS) as a true head movement [1, 32]. Cathodal galvanic stimuli depolarize, whereas anodal stimuli hyperpolarize afferent vestibular fibers [33, 34]. The unanticipated vestibular stimulus elicits a protective reflex in all muscles engaged in posture control, leading the body to temporarily sway toward the anode. The motor reflexes that are evoked to maintain the postural equilibrium can be captured by surface electromyography (EMG) in the body muscles involved in one’s posture. Galvanic-VEMP evaluates the brainstem function, as other VEMPs do [35], and further assesses the vestibulospinal motor tract. The chosen muscle to record the electrophysiological sign defines the tested level of the spine. The assessment of the spine is performed by recording the response in the sternocleidomastoid muscle for the cervical level, the trunk (erectors spinae) muscles for the thoracic level, and the lower limb muscles, such as soleus or gastrocnemius, for the lumbar spinal level [1, 32]. Graphically, the galvanic-VEMP response taken from gastrocnemius muscle is characterized by a biphasic wave, with a short-latency (SL) response around 60 ms, followed by a medium-latency (ML) response around 100 ms [1, 32, 36, 37]. A change in the waveform and the delay or the absence of any of the waves are considered altered results [2–5].

Galvanic-VEMP proved to be quite accurate in identifying spinal cord impairments based on the ROC curve in individuals with myeloradiculopathy caused by *Schistosoma mansoni* [5]. However, to the best of our knowledge, the reliability and agreement of this exam have not been checked properly in prior studies, and this assessment is essential when the exam is used for diagnostic and monitoring purposes. This study determined the interrater and the intrarater agreement and reliability of galvanic-VEMP in individuals with HAM, asymptomatic HTLV-1 infection and controls.

### The concepts and importance of agreement and reliability

The estimates of agreement (repeatability and reproducibility) and reliability are used to evaluate the measurement error of a quantity and its impact on the interpretation of measurements. Any measurement is susceptible to various types of errors that can cause the measured value to be different from the real value. Repeatability of the results (of a measurement) is the approximation between the results of successive measurements of a quantity carried out under the same measurement conditions [38]. These conditions are referred to as repeatability conditions, which include: the same measurement procedure; the same examiner (or rater); the same measuring instrument, used under the same conditions; the same place; and the repetition should be performed within a short time. Reproducibility of the results (of a measurement) is the approximation between the results of the measurements of a quantity carried out with changes in the measurement conditions [38]. Changes considered include the principle and method of measurement, the examiner, the instrument, the reference standard, the location, the conditions of use, and the time. Repeatability and reproducibility are grouped together in the concept of agreement, i.e., how far apart the repeated measures of the same quantity are. Reliability, on the other hand, correlates the magnitude of the measurement error of the repeated measurements with the inherent, error-free variability among individuals. Therefore, it depends on the variability of the population. If reliability is high, measurement errors are small relative to the actual differences among individuals in the population, and the method can differentiate well despite the measurement error [39].

The measurement error of the repeated measurements may be due to intraindividual biological variability, intrinsic variability to the measuring instrument, variability between one instrument and another, circumstances in which the measurement is performed, intrarater variability (the same examiner gives two different judgments at two different times) and interrater variability (one examiner gives a different judgment from the other examiner). By measuring and quantifying the measurement error (through repeatability, reproducibility, and reliability estimates), it is possible to judge whether this error is acceptable within the context in which the measurement is to be applied [39].

## Methods

### Ethical statement

**Fig 1:**
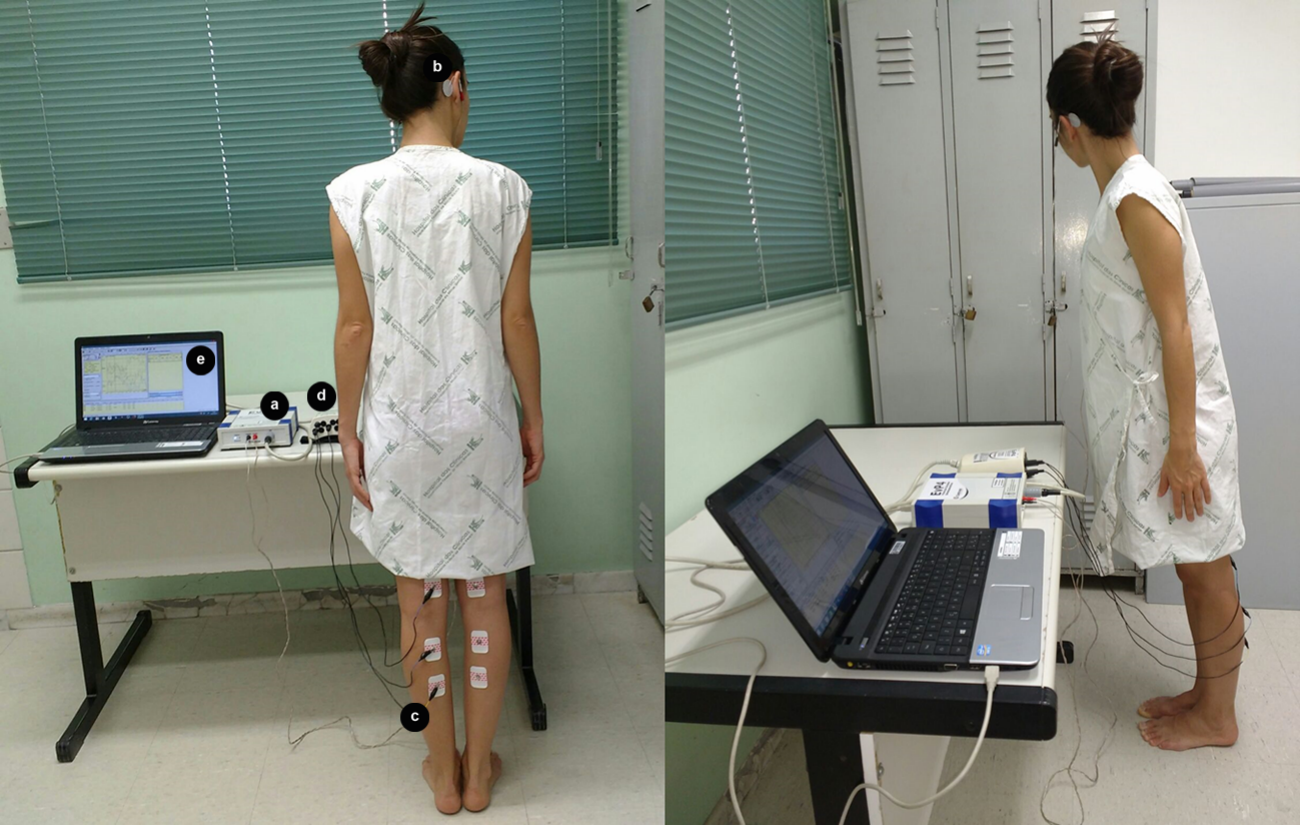
Vestibular-evoked myogenic potential triggered by galvanic vestibular stimulation procedure. The standing position of the patient (barefoot on a hard flat surface with eyes closed, feet close together and body leaning forward in order to cause the gastrocnemius muscle contraction); the equipment used for stimulus generation (a); the electrode positions for GVS (b); the electrode position for electromyography on the gastrocnemius muscle (c); the equipment for signal processing (d); and the laptop (e) connected to (a) and (d).

This study follows the ethical principles expressed in the Declaration of Helsinki [40]. It was approved by the Research Ethics Committees of Universidade Federal de Minas Gerais (UFMG) and of the Hemominas Blood Transfusion Agency, in Brazil, under the protocol numbers, respectively, of 266/05 and 131. All participants gave their written informed consent. The individual in Fig 1 has given written informed consent (as outlined in PLoS consent form) for this photograph to be published.

### Study design and setting

This is a repeatability and reproducibility study about the use of galvanic-VEMP to test HAM, which was conducted between 2014 and 2016 in the UFMG School of Medicine, Belo Horizonte, Brazil.

### Subjects and sample size

The individuals were recruited from the open cohort of the Interdisciplinary HTLV Research Group (GIPH), formed in 1997, which has been following the individuals from 1997 to the present day [41–44]. The inclusion criteria for the infected individuals were positive serology in Enzyme-linked Immunosorbent Assay and Western Blot, as well as positive Protein Chain Reaction, for HTLV-1. The HTLV-1 infected individuals are divided into asymptomatic carriers (AC) and individuals with HAM, according to the revised diagnostic criteria by Castro-Costa et al. [45]. The controls tested negative for HTLV-1. The exclusion criteria for all groups were: under 18 years of age, uncontrolled acute or chronic diseases, HIV coinfection, suspected or confirmed pregnancy, metallic prosthesis, being unable to stand in the upright position during the galvanic-VEMP procedures, neurologic diagnosis such as history of stroke, CNS tumor, CNS infection (other than HTLV-1 infection), vitamin B12 deficiency, spinal cord diseases (other than HAM), diabetic neuropathy, migraine, and, finally, vestibular diseases such as Benign Paroxysmal Positional Vertigo (BPPV), vestibular neuritis and Ménière’s disease. All the subjects were submitted to a clinical interview and neurological examination before undergoing galvanic-VEMP procedures.

Considering the study by Shoukri et al. (2004) [46], for a repeatability study to achieve reliable results with two repeated measurements, a significance level of 5%, and a test power of 80%, a minimum sample of 86 participants is necessary. In the present study, the total sample included 96 participants. Since the interest variables (SL and ML) were collected from both legs of each individual, a randomization, performed by the statistical computer program, was performed to select which leg of each participant would be part of the analyses.

### Measurement process

#### Technical aspects and protocol of the galvanic-VEMP

The galvanic-VEMP equipment used for stimulation and recording was the EvP4 / ATCPlus model (Contronic Ltda., Pelotas, Brazil) connected to a battery-powered portable computer. Self-adhesive surface electrodes, 3 centimeters (cm) in diameter (model CF3200-Valutrode, Axelgaard, Fallbrook, CA, USA) were positioned on the participant’s mastoid processes, anode on one side and cathode on the other, offering bipolar binaural stimulation. The stimulus was generated by a constant current stimulator, consisting of a single-phase, rectangular, direct current with an intensity of 2 mA for 400 ms [3–5].

Each examination consisted of 30 stimulations, 15 of which were performed with the anode in the right ear and 15 with anode in the left ear. Intervals between the stimuli were randomized between 4 and 6 seconds. The test was then immediately repeated once to evaluate repeatability.

To perform the test, the subjects stood on a hard flat surface with their eyes closed, barefoot, with their body slightly bent forward, promoting contraction of the gastrocnemius muscle. Participants were instructed to turn their heads approximately 90° in the sagittal plane to the contralateral side of the lower limb from which the EMG signals would be drawn [36].

The EMG activity was recorded by a pair of self-adhesive electrodes (model 2223BRQ, 3M, Saint Paul, MN, USA) placed on the medial head of the gastrocnemius muscle, and with their centers approximately 5 cm distant from one another. The reference electrode was placed on the back of the thigh, approximately 5cm above the recording electrode (Fig 1). Galvanic-VEMP was performed first on the left lower limb and then on the right lower limb. Performing the complete examination of a patient lasted about 20 minutes on average.

The EMG signals were measured, rectified, filtered between 10 Hertz (Hz) and 1000 Hz, and scanned at a sampling frequency of 5 kHz, using one register channel. The data were collected during a period of 500 ms, beginning at 100 ms before the galvanic stimulus [4]. The EMG responses to 15 consecutive stimuli with the same polarity setting were averaged, resulting in a final tracing. The tracings could be observed online during the execution of the exam and were recorded for further analysis by the examiners, under blindness as to the group to which the participant belonged.

#### Definition of the galvanic-VEMP variables

**Fig 2:**
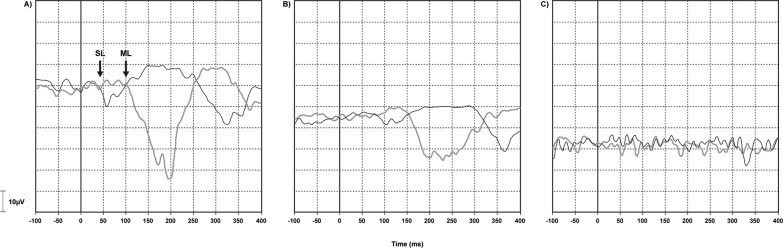
Normal, delayed, and abnormal response patterns in vestibular-evoked myogenic potential triggered by galvanic vestibular stimulation (galvanic-VEMP). (A) Normal electromyographic (EMG) response recorded from the gastrocnemius muscle. The black line indicates the trace with the cathode on the right and the anode on the left, whereas the gray line indicates the opposite stimulation polarity. SL (~50 ms) and ML (~100 ms). (B) Delayed EMG responses. SL ~80 ms and ML ~150 ms. (C) Absent EMG response, no SL and no ML.

The measured variables were the latencies of each of the two components of the galvanic-VEMP wave. The rater analysis was based on previously described criteria [36, 47], i.e.: SL is the wave starting at about 60 ms and ML is the following wave, with opposite polarity, starting at about 100 ms. SL and ML reverse with the inversion of stimulus polarity. The responses were considered to be changed if they were delayed, absent, or with abnormal tracing. Delay was considered when response onset was later than 2 standard-deviations over the mean found in healthy controls [4], i.e., 63 ms for SL and 132 ms for ML. Fig 2 illustrates the normal, delayed, and abnormal tracing patterns.

For a more detailed description of this test, go to dx.doi.org/10.17504/protocols.io.nxbdfin.

### Statistical analysis, agreement and reliability parameters

This study analyzed the agreement and reliability of measurements of the EMG responses of galvanic-VEMP. For each of the two EMG responses (SL and ML), the estimates were calculated based on two measurements done (a) in repeatability conditions, i.e., two immediately repeated measurements in the same patient, analyzed by the same examiner–test-retest repeatability or intrarater repeatability (b) by two experienced independent examiners, blinded to the clinical condition of the participant–interrater reproducibility.

The calculated agreement parameters included: standard error of measurement (SEM) = SD of the paired differences / √2, within-individual variation, limits of agreement, and the coefficient of repeatability (CR) = SD of paired differences x 1.96. SEM and CR represent the measurement error intrinsic to the measurement method and take into consideration the within-subject variation. The CR shows the expected variation of the results for 95% of the repeated measures, which is expressed in the same unit of measure. It is also known as the Smallest Real Difference (SRD).

The reliability of the test was assessed by the intraclass correlation coefficient (ICC) and the Kappa coefficient. ICC indicates good reliability when equal to or higher than 0.70, [39, 48]. The Kappa coefficient was considered acceptable if greater than 0.6 [49]. The Kappa coefficient was calculated after the categorization of the variables into normal, delayed, and absent, following the criteria described in the previous section.

The database was fed with double input using the EpiData 3.0 program (EpiData Data Entry, Data Management and basic Statistical Analysis System, EpiData Association, 2000–2008, Odense, Denmark). The SPSS 15.0 program (SPSS, Inc., Chicago, IL, USA) was used to describe the variables and conduct statistical analysis. Continuous variables of interest were tested for normality with Shapiro-Wilk test and showed a non-normal distribution. The non-parametric Kruskal-Wallis test was used to compare continuous variables between groups. For categorized variables, a chi-square test (Pearson’s or Fisher’s) was used. The significance level was 5%.

## Results

From a total of 100 individuals selected for the study, four were excluded: one reported a metal plaque implant in the skull, one had HIV infection, and we lost the galvanic-VEMP tracings in two patients due to interference in the software device. Of the 96 participants who completed the entire protocol, 45 were controls 27 were asymptomatic HTLV-1 carriers (AC), and 24 were individuals with HAM. The mean age was 55, 58, and 58 years in the control, AC, and HAM groups, respectively, with no statistical difference (p = 0.552). The proportion of male gender was 40, 41, and 29 percent in the control, AC, and HAM groups, respectively, with no statistical difference (p = 0.624). The comparison of the continuous and categorized (normal, delayed, or absent) galvanic-VEMP responses (SL and ML) are shown in Table 1. In the HAM group, the SL showed a tendency toward higher values (p = 0.089) and was more frequently delayed and absent (p = 0.067). The ML was delayed and more frequently absent in the HAM group when compared to the AC and control groups (p<0.001).

**Table 1:** Galvanic-VEMP variables (SL and ML): Comparison between groups.

Variable		HTLV-1 negative controls (n = 45)	Asymptomatic carriers (n = 27)	HAM (n = 24)	p-value
SL	Median (IQR)	56 (53–64)	53 (52–63)	63 (56–75)	0.089
	normal	29(64.4%)	14 (51.9%)	8 (33.3%)	0.067
	delayed	10 (22.2%)	4 (14.8%)	8 (33.3%)	
	absent	6 (13.4%)	9 (33.3%)	8 (33.3%)	
ML	Median (IQR)	114 (105–126)	116 (101–130)	136 (124–144)*	<0.001
	normal	42 (93.3%)	17 (63%)	8 (33.3%)	<0.001
	delayed	2 (4.4%)	4 (14.8%)	11 (45.8%)	
	absent	1 (2.3%)	6 (22.2%)	5 (20.9%)	

Notes: SL delay: > 63ms; ML delay: > 132ms; IQR: interquartile range. Statistical tests: Kruskal-Wallis for continuous SL and ML values; Qui-square for categorized SL and ML.

* statistically different group.

### Agreement and reliability of the galvanic-VEMP responses (SL and ML)

The agreement and the reliability measures were acceptable in intrarater (test-retest) and interrater calculations for SL and ML in the total sample and in each group (Tables 2–5). There was no clinically relevant difference of these parameters between the groups.

**Table 2:** Intrarater (test-retest) and interrater agreement and reliability measures of galvanic-VEMP variables (SL and ML) in the total sample (n = 96).

	Variable	SEM	CR	ICC	P value	Kappa	P value
Intrarater	SL	6	16	0.803	<0.001	0.533	<0.001
	ML	8	22	0.913	<0.001	0.829	<0.001
Interrater	SL	3	8	0.953	<0.001	0.769	<0.001
	ML	10	27	0.863	<0.001	0.884	<0.001

SEM: standard error of measurement. CR: coefficient of repeatability. ICC: intraclass correlation coefficient.

**Table 3:** Intrarater (test-retest) and interrater agreement and reliability measures of galvanic-VEMP variables (SL and ML) in HTLV-1 negative controls (n = 45).

	Variable	SEM	CR	ICC	P value	Kappa	P value
Intrarater	SL	5	14	0.688	<0.001	0.494	<0.001
	ML	7	19	0.860	<0.001	0.567	<0.001
Interrater	SL	2	5	0.963	<0.001	0.587	<0.001
	ML	12	33	0.752	<0.001	0.395	<0.001

SEM: standard error of measurement. RC: repeatability coefficient. ICC: intraclass correlation coefficient.

**Table 4:** Intrarater (test-retest) and interrater agreement and reliability measures of galvanic-VEMP variables (SL and ML) in HTLV-1 asymptomatic carriers (n = 27).

	Variable	SEM	CR	ICC	P value	Kappa	P value
Intrarater	SL	6	16	0.694	0.014	0.567	<0.001
	ML	8	23	0.923	<0.001	0.861	<0.001
Interrater	SL	4	12	0.861	<0.001	0.878	<0.001
	ML	4	12	0.946	<0.001	0.749	<0.001

SEM: standard error of measurement. CR: coefficient of repeatability. ICC: intraclass correlation coefficient.

**Table 5:** Intrarater (test-retest) and interrater agreement and reliability measures of galvanic-VEMP variables (SL and ML) in individuals with HAM (n = 24).

	Variable	SEM	CR	ICC	P value	Kappa	P value
Intrarater	SL	7	19	0.850	0.001	0.438	0.002
	ML	10	29	0.861	<0.001	0.869	<0.001
Interrater	SL	3	8	0.978	<0.001	0.813	<0.001
	ML	9	25	0.808	0.001	0.509	<0.001

SEM: standard error of measurement. CR: coefficient of repeatability. ICC: intraclass correlation coefficient.

## Discussion

Galvanic-VEMP has been used to investigate the postural balance in normal individuals for more than four decades [50–55], and in recent years this exam has been considered to be an auxiliary tool for the diagnosis of myelopathies [2–5]. The accuracy of galvanic-VEMP has been described [5], but not the agreement and reliability, which are equally important to validate a diagnostic tool.

The present study evaluated, for the first time, the agreement and the reliability of galvanic-VEMP between two repeated measurements (intrarater test-retest) and between measurements made by two examiners (interrater). Galvanic-VEMP is a test that measures the time, in milliseconds, of a postural reflex from its generation by electric stimulation of the vestibular nuclei until its muscular response, which is recorded by surface electromyography. Therefore, the response can be recorded only from the muscles involved in the balance control.

Several factors can lead to a variability / measurement error of galvanic-VEMP latencies: 1) the circadian biological variations of individuals; 2) possible intrinsic instabilities of the devices; 3) the variability in the interpretation of the examiner when analyzing the electromyography curve; 4) the variability of interpretation of different examiners; 5) the variability of sensory perception, such as vision, hearing, and proprioception, which influences the EMG responses. Aimed at reducing external bias, the test is conducted in a silent environment, with a grounded electrical grid, and the patient must be able to maintain a correct posture during the exam, with eyes closed [36, 45, 56–58].

A practical way for clinicians to evaluate the error of measurement (both random and systematic errors) is by observing the CR, which is expressed in the same unit as the measurement tool (in milliseconds, in our case). It is expected that the absolute difference between two measurements on a subject differs by no more than the repeatability coefficient in 95% of the occasions. For this reason, the CR is also referred to as the Smallest Real Difference (SRD) [59]. In our results, CR was 16 ms for SL and 22 ms for ML in intrarater repeated measures, and 8 ms for SL and 27 ms for ML in interrater repeated measures, meaning that latency differences larger than these values are due to real differences and not measurement errors, considering a 95% probability. These estimates are important to be considered when the method is going to be used to detect the real difference within-subject in the disease progression or therapeutic response, which are, for instance, the proposed uses for galvanic-VEMP. The CR is calculated based on the standard error of measurement (SEM). SEM alone can be interpreted when there is an established concept of the differences that are clinically relevant. However, regarding the variables SL and ML, there is still no conclusion about how large the difference must be in order to be considered a significant change in the exam. In our study, SEM was 6 ms (intrarater) and 3 ms (interrater) for SL and 8 ms (intrarater) and 10 ms (interrater) for ML. As far as we know, the only available reference parameters are from two cross sectional studies. Cunha et al. found that SL was 67±8 ms in the group with HAM and 55±4 ms in the controls–a difference of 12 ms between the means, while ML was 130±3 ms in HAM and 112±10 in controls–a difference of 18 ms between the means [4]. In patients with schistosomal myeloradiculopathy the SL was 64 ms (60/74) and 59 ms (56/61) in the controls–a difference of 5 ms between the medians, while the ML was 138 ms (122/153) in patients and 109 ms (106/121) in controls–showing a larger difference of 29 ms [5]. Longitudinal studies with larger samples are warranted to define the clinically relevant change in SL and ML when monitoring HAM and other myelopathies.

For the risk prediction and the diagnosis, on the other hand, it is essential to determine if, despite the error, the method can distinguish the individuals, taking into consideration the variability between people. This aspect is linked to reliability and is assessed by the intra-class correlation coefficient (ICC) [46, 47]. A good ICC is considered to be ≥ 0.70, which means that at least 70% of the variability in measurements is estimated to be due to real differences in the values, with the remaining 30% or less being due to errors in the measurement process [46, 47]. In our study, galvanic-VEMP proved to be reliable, with very good ICCs: 0.803 (SL) and 0.913 (ML) for intrarater measurements pairs and 0.953 (SL) and 0.863 (ML) for interrater pairs.

The agreement and the reliability parameters described above are suitable for continuous variables. To include galvanic-VEMP in the battery to test the postural reflex, the responses must be categorized into normal, delayed, and absent (criteria described in the methods section). For the categorized results, we calculated the Kappa coefficient, which proved to be quite satisfactory for ML in intrarater and interrater analyses (greater than 0.80). For SL, the interrater Kappa was good (0.769), but for intrarater repeated measurements, it was not clinically acceptable (0.533). The Kappa was under the acceptance level especially in the control group. However, one limitation is that the normality cutoffs used in our study were based on the results of normal individuals from a study with a sample of 13 subjects [4], i.e. we considered the normality cutoff as being 2 standard-deviations over the mean found in this healthy small group. The ROC curve of the galvanic-VEMP showed good results (0.814 for SL, p = 0.001, and 0.861 for ML, p<0.001) in a study with schistosomal myeloradiculopaty [5], but the cutoff values of SL and ML were not described. Therefore, future studies on accuracy for definition of normality cutoffs should be conducted.

## Conclusion

Galvanic-VEMP proved to have good accuracy [5], and the present results also show good repeatability, reproducibility, and reliability. For the time being, there is still no definition if a change in galvanic-VEMP in HTLV-1-asymptomatic carriers is a biomarker of HAM. A longitudinal study will fill this knowledge gap. We conclude that this test can be considered for the follow-up of HAM, since it proved to be a reliable low-cost, easy to perform, and safe tool to test the postural reflex.

## Supporting information

S1 Table Abbreviations meaning. (DOCX)

S1 Appendix GRRAS checklist for reporting of studies of reliability and agreement. (PDF)

S1 Dataset (SAV)
