# Supplementary material for: Intrarater and interrater agreement and reliability of vestibular evoked myogenic potential triggered by galvanic vestibular stimulation (galvanic-VEMP) for HTLV-1 associated myelopathy testing
Source: PLoS One. 2018 Sep 27;13(9):e0204449. doi: 10.1371/journal.pone.0204449 (PMC6160040; doi:10.1371/journal.pone.0204449)
Supplement: S1 Table — (DOCX) [file pone.0204449.s001.docx]

**S1 Table. Abbreviations meaning.**

| **AC** | asymptomatic carriers |
| --- | --- |
| **cm** | centimeters |
| **CNS** | central nervous system |
| **CR** | coefficient of repeatability (also known as smallest real difference - SRD) |
| **EMG** | electromyography |
| **Galvanic-VEMP** | vestibular evoked myogenic potential triggered by galvanic vestibular stimulation |
| **GIPH** | Interdisciplinary HTLV Research Group |
| **GVS** | galvanic vestibular stimulation |
| **HAM** | HTLV-1 associated myelopathy |
| **HTLV-1** | human t-cell lymphotropic virus type 1 |
| **Hz** | Hertz |
| **ICC** | intraclass correlation coefficient |
| **IQR** | interquartile range |
| **kHz** | kilohertz |
| **mA** | milliamperes |
| **ML** | medium latency response |
| **ms** | milliseconds |
| **ROC** | receiver operating characteristic |
| **SD** | standard deviation |
| **SEM** | standard error of measurement |
| **SL** | short latency response |
| **SRD** | smallest real difference (also known as coefficient of repeatability - CR) |
| **UFMG** | Universidade Federal de Minas Gerais |
| **VEMP** | Vestibular evoked myogenic potential |
