# Supplementary material for: Intrarater and interrater agreement and reliability of vestibular evoked myogenic potential triggered by galvanic vestibular stimulation (galvanic-VEMP) for HTLV-1 associated myelopathy testing
Source: PLoS One. 2018 Sep 27;13(9):e0204449. doi: 10.1371/journal.pone.0204449 (PMC6160040; doi:10.1371/journal.pone.0204449)
Supplement: S1 Appendix — (PDF) [file pone.0204449.s002.pdf]

GRRAS checklist for reporting of studies of reliability and agreement

Version based on Table I in: Kottner J, Audigé L, Brorson S, Donner A, Gajeweski BJ, Hróbjartsson A, Robersts C, Shoukri M, Streiner DL. Guidelines for reporting reliability and agreement studies (GRRAS) were proposed. J Clin Epidemiol. 2011;64(1):96-106

**Section**	**Item #**	**Checklist item**	**Reported on page #**
Title/Abstract	1	Identify in title or abstract that interrater/intrarater reliability or agreement was investigated.	✓
Introduction	2	Name and describe the diagnostic or measurement device of interest explicitly.	✓
	3	Specify the subject population of interest.	✓
	4	Specify the rater population of interest (if applicable).	✓
	5	Describe what is already known about reliability and agreement and provide a rationale for the study (if applicable).	✓
Methods	6	Explain how the sample size was chosen. State the determined number of raters, subjects/objects, and replicate observations.	✓
	7	Describe the sampling method.	✓
	8	Describe the measurement/rating process (e.g. time interval between repeated measurements, availability of clinical information, blinding).	✓
	9	State whether measurements/ratings were conducted independently.	✓
	10	Describe the statistical analysis.	✓
Results	11	State the actual number of raters and subjects/objects which were included and the number of replicate observations which were conducted.	✓
	12	Describe the sample characteristics of raters and subjects (e.g. training, experience).	✓
	13	Report estimates of reliability and agreement including measures of statistical uncertainty.	✓
Discussion	14	Discuss the practical relevance of results.	✓
Auxiliary material	15	Provide detailed results if possible (e.g. online).	✓
